# Editorial: Advanced breeding for abiotic stress tolerance in crops, volume II

**DOI:** 10.3389/fpls.2026.1835864

**Published:** 2026-04-21

**Authors:** Meng Jiang, Tianlun Zhao, Qian-Hao Zhu, Xinyang Wu

**Affiliations:** 1 Hainan Institute, Zhejiang University, Sanya, China; 2 The Advanced Seed Institute, Zhejiang University, Hangzhou, China; 3 Department of Agriculture and Food, Commonwealth Scientific and Industrial Research Organisation (CSIRO), Canberra, ACT, Australia; 4 College of Life Sciences, China Jiliang University, Hangzhou, China

**Keywords:** abiotic stress, advanced techniques, crop breeding, gene family, machine learning, mechanism, multiomics analysis, QTL mapping

## 1 Introduction

Abiotic stresses, encompassing extreme temperatures (heat, cold), salinity, drought, and heavy metal toxicity, pose severe threats to global agricultural productivity and food security (Guan et al., 2025; Wang et al., 2025; Yang et al., 2024; Zeng et al., 2025). With the intensifying impacts of climate change, the frequency and intensity of these stresses are rising, necessitating urgent and advanced strategies for breeding climate smart crop varieties (Song et al., 2025; Wei et al., 2025; Zhu et al., 2025). Building upon the foundation laid in the previous volume, this Research Topic aims to further deepen our understanding of the complex molecular mechanisms governing abiotic stress tolerance in crops, so a multifaceted strategy integrating physiological, biochemical, and molecular insights can be developed to breed abiotic stress resilient crop varieties.

This Research Topic features 26 articles (22 Original Research, 4 Reviews) that collectively push the boundaries of abiotic stress research. Rather than viewing stress tolerance as isolated physiological responses, the works presented here converge on a central theme: the integration of multi-scale biological data—from single-gene functions to genome-wide networks and field-level genotype-by-environment (G×E) interactions—to engineer resilient crops.

## 2 Converging signaling pathways: from stress perception to hormonal crosstalk

A prominent cross-cutting advance in this Research Topic is the deeper understanding of how plants perceive and transduce complex stress signals, particularly highlighting the crosstalk between distinct abiotic cues. For instance, while extreme temperatures and osmotic stresses (drought/salinity) trigger distinct early responses, the downstream regulatory networks heavily overlap.

Reviews by Kumar et al. and Peng et al. synthesize how heat and cold stresses, respectively, converge on conserved transcriptional regulators (e.g., CBF/DREB1) and are modulated by emerging factors like the plant microbiome. At the molecular level, original research reveals shared signaling hubs across stress types. Yu et al. demonstrate that in wheat, the ERF transcription factor TaERF13-2B negatively regulates drought tolerance by interacting with TaCIPK9, a nexus for calcium signaling. Similarly, Tang et al. show that osmotic stresses upregulate *TaPSK* genes to promote root growth, highlighting the phytohormone-ROS axis as a universal regulatory module. Collectively, these studies shift the paradigm from viewing stress signaling in silos to mapping a highly interconnected phytohormone and calcium-dependent network.

## 3 Expanding the regulatory lexicon: beyond protein-coding genes

Traditionally, abiotic stress tolerance research has focused on functional protein-coding genes. This Research Topic significantly broadens that scope by emphasizing the roles of epigenetic regulation, non-coding RNAs, and previously underappreciated gene families.

Several studies systematically map the landscape of stress-responsive gene families across diverse crops (e.g., WAKs in tobacco by Li et al., ZIPs in foxtail millet by Zheng et al., and JRLs in gramineous plants by Gong et al.), establishing a rich genomic resource. However, the true mechanistic breakthroughs lie in the layers of regulation above the genome. Wang et al. uncover the role of *TaALKBH9B-5* in m6A RNA methylation under cold stress in wheat, while Zhao et al. identify a long non-coding RNA (*TalncRNA18313*) that enhances cadmium tolerance by modulating antioxidative defense. By integrating these layers, this Research Topic illustrates that stress resilience is not solely determined by genetic sequence, but by dynamic transcriptomic and epigenetic remodeling.

## 4 Bridging the lab-to-field gap: QTL discovery, multi-omics, and AI-driven breeding

Perhaps the most translational contribution of this Research Topic is the methodological shift from candidate gene validation to predictive, data-driven breeding. Identifying causal genes is only the first step; integrating them into breeding pipelines requires advanced phenotyping and environmental modeling.

This Research Topic excels in linking genetic loci to agronomic traits. Jo et al. fine-map the stable cold-tolerance QTL *qCTS1022/23* in rice, while Xu et al. utilize GWAS to identify a negative regulator of salt tolerance (*LOC_Os02g36880*) in Japonica rice. Zhang et al. further translate allelic variations in wheat *TaMFT-3A* and *TaMKK3-4A* into practical markers for pre-harvest sprouting resistance.

Crucially, these genetic discoveries are paired with cutting-edge agronomic tools. Wang et al. develop a machine learning model (Random Forest) integrating meteorological data to predict maize hybrid yields, while Yue et al. employ environmental classification and AMMI models to dissect G×E interactions in winter wheat. These studies demonstrate a complete translational pipeline: from multi-omics network analysis (e.g., Tian et al. using WGCNA for xylem plasticity in mulberry) to algorithmic yield prediction under stress.

## 5 Conclusion and collective contributions

In summary, this Research Topic transcends a mere cataloging of stress-responsive genes. The collective contribution of these 26 articles lies in their demonstration of a holistic, multi-omics framework for crop improvement. They collectively argue that future breakthroughs in abiotic stress tolerance will not rely solely on single-gene overexpression, but on the precise manipulation of regulatory networks (epigenetics, lncRNAs), the strategic pyramiding of broad-spectrum QTLs, and the application of machine learning to navigate complex G×E interactions. By synthesizing these diverse approaches, this Research Topic provides a concrete roadmap for moving from molecular mechanisms to the delivery of climate-smart crop varieties.
